# A Rare Presentation of Cecal Adenocarcinoma Causing Adult Ileocolic Intussusception: A Case Report

**DOI:** 10.7759/cureus.108018

**Published:** 2026-04-30

**Authors:** Maria Fioletova, Nicholas Riemen, Tyler Allsage, Rachel Rauber, James Doty

**Affiliations:** 1 General Surgery, Nova Southeastern University Dr. Kiran C. Patel College of Osteopathic Medicine, Davie, USA; 2 General Surgery, Nova Southeastern University Dr. Kiran C. Patel College of Allopathic Medicine, Fort Lauderdale, USA; 3 General Surgery, HCA Florida Westside Hospital, Plantation, USA

**Keywords:** adult intussusception, cecal adenocarcinoma, colorectal cancer, ileocolic intussusception, pathological lead point

## Abstract

Intussusception occurs when a segment of the bowel telescopes into an adjacent segment, potentially leading to obstruction and intestinal ischemia. Intussusception in adults is likely caused by a pathological lead point (PLP), and it can be challenging to diagnose due to its broad clinical presentation, which requires a high degree of clinical suspicion. This case report describes a rare presentation of cecal adenocarcinoma in a young patient that caused ileocolic intussusception. A 31-year-old woman presented to the emergency department with progressively worsening intermittent abdominal pain, nausea, and bloating. She denied fever, chills, vomiting, hematochezia, and unintentional weight loss. Computed tomography (CT) scan of the abdomen and pelvis suggested ileocecal intussusception and two irregular cystic masses in the liver. After a colonoscopy showed a necrotic intraluminal mass, the patient underwent an exploratory laparotomy with right hemicolectomy due to the high risk of malignancy-associated intussusception. Pathology results from the hemicolectomy were consistent with adenocarcinoma of colonic origin. Furthermore, a liver biopsy was conducted to confirm metastases. The recovery was uncomplicated, with a gradual return of bowel function and tolerance of oral intake. A central venous port was placed for anticipated systemic chemotherapy, and the patient was referred for outpatient oncology follow-up, including positron emission tomography (PET)-CT imaging and initiation of chemotherapy. This case highlights the importance of considering colorectal malignancy in the differential diagnosis of young adults presenting with nonspecific gastrointestinal symptoms. Early diagnosis with high-contrast CT imaging is critical for identifying intussusception, evaluating for PLPs, and detecting metastatic disease, while prompt surgical intervention remains essential given the rarity of adult intussusception and the high likelihood of an underlying malignant etiology.

## Introduction

Intussusception is characterized by one bowel segment telescoping into an adjacent segment, which can cause obstruction and even intestinal ischemia [1]. Intussusception is more common in children due to idiopathic causes, possibly due to viral infections, and in adults, it is most frequently associated with colon cancer [2]. It is challenging to diagnose due to its broad, nonspecific complaints and requires a high level of clinical suspicion. Potential warning signs of colorectal cancer in younger adults that have been more commonly reported are abdominal pain, rectal bleeding, diarrhea, and iron deficiency anemia [3]. When intussusception does occur, it is likely caused by a pathological lead point (PLP), such as a malignant growth from cancer. A 2006 study confirmed that intussusception is a statistically significant predictor of malignancy until proven otherwise [4]. Pooled data from a large meta-analysis report an overall malignancy rate of 32.9% in adult intussusception, with higher rates observed in colonic cases [5].

In the United States, colorectal cancer is the second most common cause of cancer death diagnosed in adults aged 50 and older. It has a lower survival rate than other types of cancer, affected by variables such as age, socioeconomic status, and tumor stage. More specifically, cecal carcinoma is a common malignant tumor with high mortality, which accounts for approximately 20% of colorectal cancers [6]. In a cohort study conducted in 2014, patients with right-sided colorectal cancer were associated with larger tumor size, poorer differentiation, more advanced stage distribution, and greater lymph node involvement compared with left-sided tumors, although differences in disease-free survival were inconsistent across tumor stages [7]. These patterns likely reflect underlying molecular differences, with right-sided tumors more often linked to germline mutations in advanced stages and higher recurrence rates compared to patients with left-sided colorectal cancer, which are more likely to be sporadic.

The main goal is to describe a rare presentation of cecal adenocarcinoma in a young patient that caused ileocolic intussusception. The significance is to maintain a high index of suspicion for colorectal cancer in young patients, particularly those without colorectal cancer family history or those presenting with nonspecific abdominal symptoms or bowel obstructions. Furthermore, this study highlights the diagnostic value of imaging and the need for prompt surgical intervention. This report also aims to increase clinical awareness, earlier diagnosis, and ultimately improve outcomes in similar atypical presentations.

## Case presentation

A 31-year-old woman presented to the emergency department with five days of progressively worsening intermittent abdominal pain, nausea, bloating, and hyperactive bowel sounds. The patient had a past medical history of migraine and had had two episodes of melena and one episode of dark, purplish stool. She denied fever, vomiting, hematochezia, dysuria, or weight loss. There was no history of previous colonoscopy or of colorectal cancer in the family; however, her mother had breast cancer, and her aunt had ovarian cancer.

**Figure 1:**
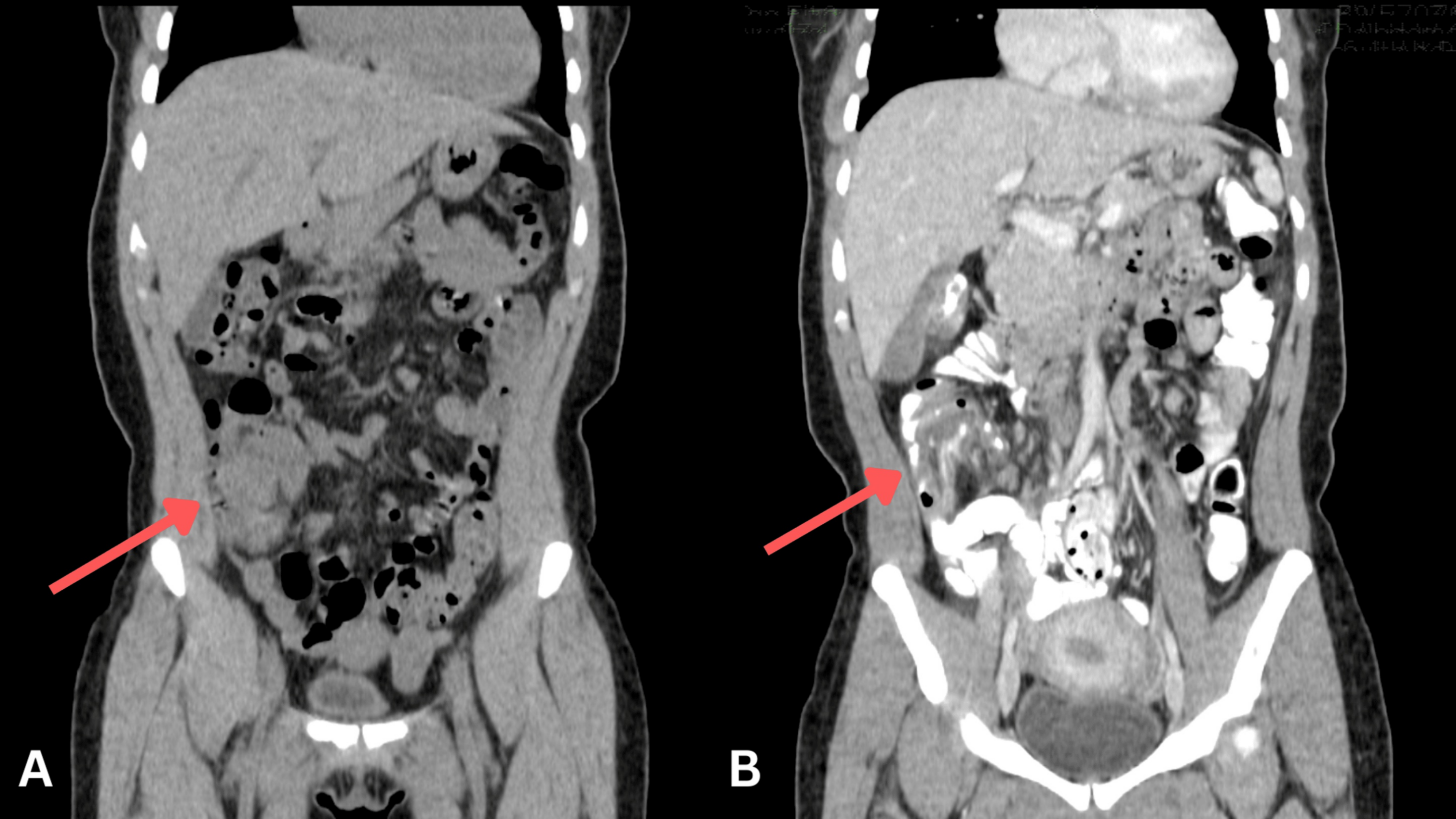
CT image of the abdomen and pelvis, coronal view, no contrast (A). CT image of the abdomen and pelvis, coronal view, with contrast (B). Red arrows point at ileocolic intussusception without contrast (A) and with contrast (B). CT: computed tomography

**Figure 2:**
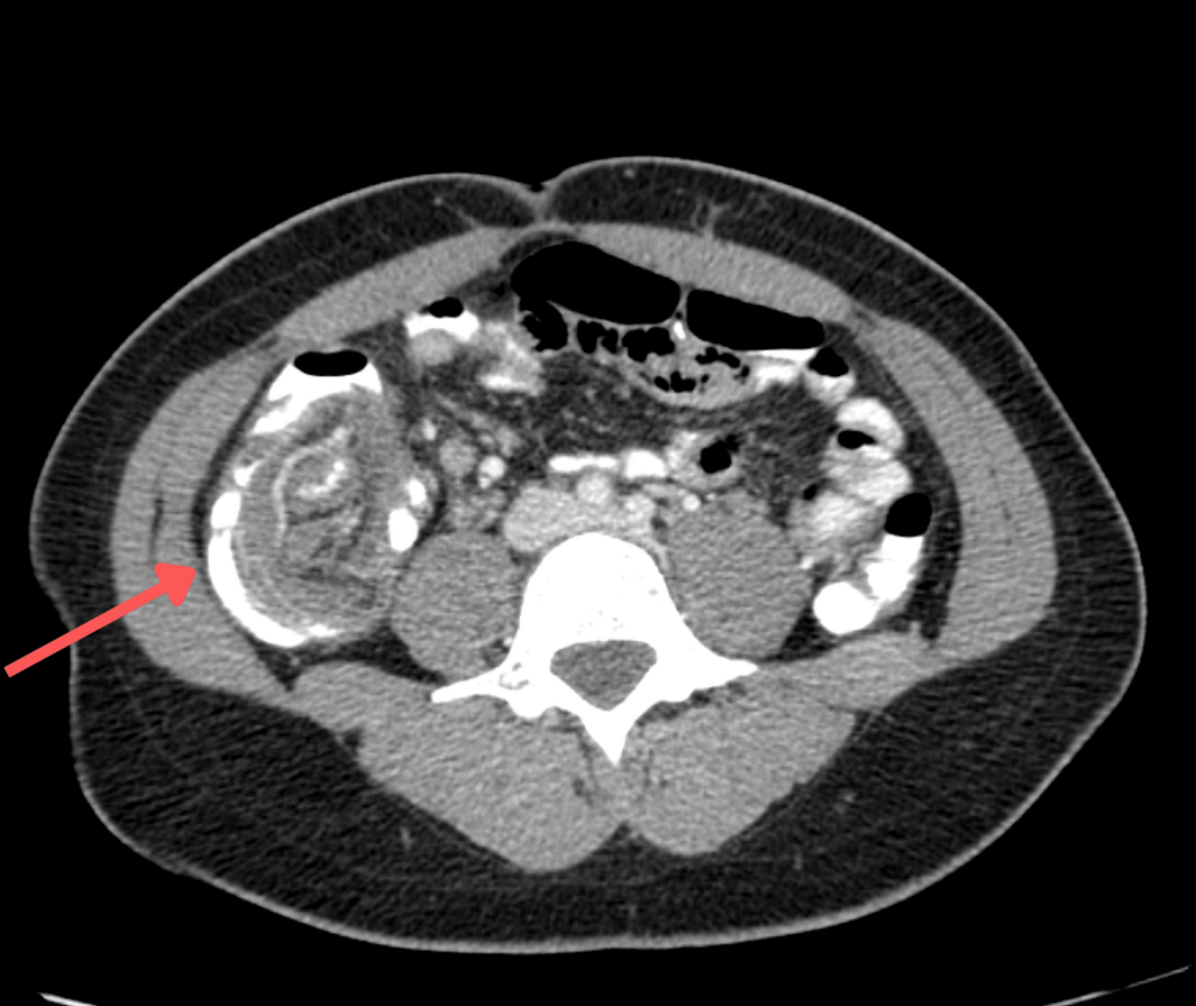
CT image of the abdomen and pelvis, cross-sectional view, with contrast. The red arrow points at an ileocolic intussusception. CT: computed tomography

**Figure 3:**
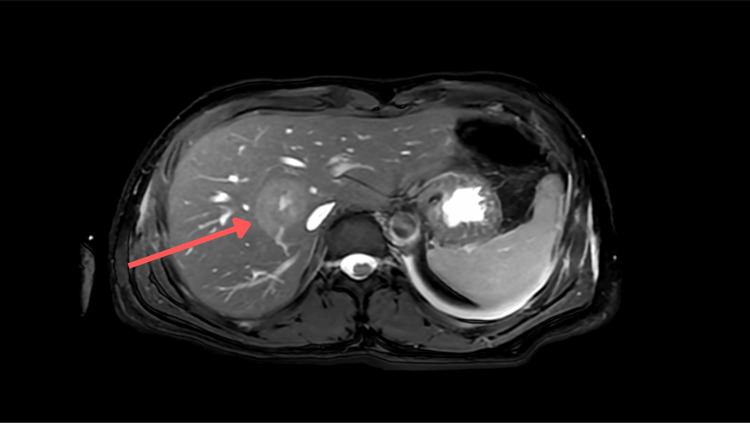
CT image of the liver showing metastases. The red arrow points at liver metastases. CT: computed tomography

On presentation, the patient was hemodynamically stable. The physical examination demonstrated a soft, non-distended abdomen without rebound tenderness. Laboratory evaluation revealed no leukocytosis, though subsequent testing demonstrated normocytic anemia (hemoglobin of 11.7 g/dL, serum iron of 70 µg/dL, total iron-binding capacity (TBIC) of 320 µg/dL, iron saturation of 22%, ferritin of 13.7 ng/mL), consistent with iron deficiency. Initial computed tomography (CT) of the abdomen and pelvis without contrast demonstrated findings concerning for ileocolic intussusception with mild appendiceal prominence, prompting repeat imaging with oral contrast (Figure 1). Repeat CT imaging of the abdomen and pelvis, performed on hospital day 2, confirmed the original diagnosis of ileocolic intussusception with irregular mucosa at the upper margin, raising concerns for a PLP (Figures 1, 2). Additionally, two irregular cystic lesions in the liver were identified, which raised concerns for metastatic disease (Figure 3). In this case, contrast-enhanced CT was critical in identifying ileocolic intussusception with irregular mucosa concerning malignancy, as well as hepatic lesions suspicious for metastases, prompting early multidisciplinary involvement. Moreover, serum tumor markers were elevated, with carcinoembryonic antigen (CEA) at 8.6 and carbohydrate antigen (CA) 19-9 at 191.

Despite the imaging findings, the patient remained clinically stable, with intermittent abdominal discomfort but no signs of acute obstruction or peritonitis, continuing to pass flatus with a non-distended abdomen. Further diagnostic evaluation was conducted to confirm the findings of adult intussusception, gastrointestinal bleeding, anemia, and imaging features suspicious for malignancy. These were treated with endoscopic and surgical intervention, as described in the next sections.

Management

Given the imaging findings and concern for malignancy, the patient was admitted for further evaluation. Initial management included bowel rest, intravenous fluids, and analgesia, with serial abdominal exams. Although hepatic lesions suspicious for metastases were identified on imaging, their presence did not preclude surgical resection of the primary tumor. Given the risk of progression to complete obstruction, delaying surgery for preoperative biopsy was not pursued. General surgery and gastroenterology were consulted early in the hospital course.

**Figure 4:**
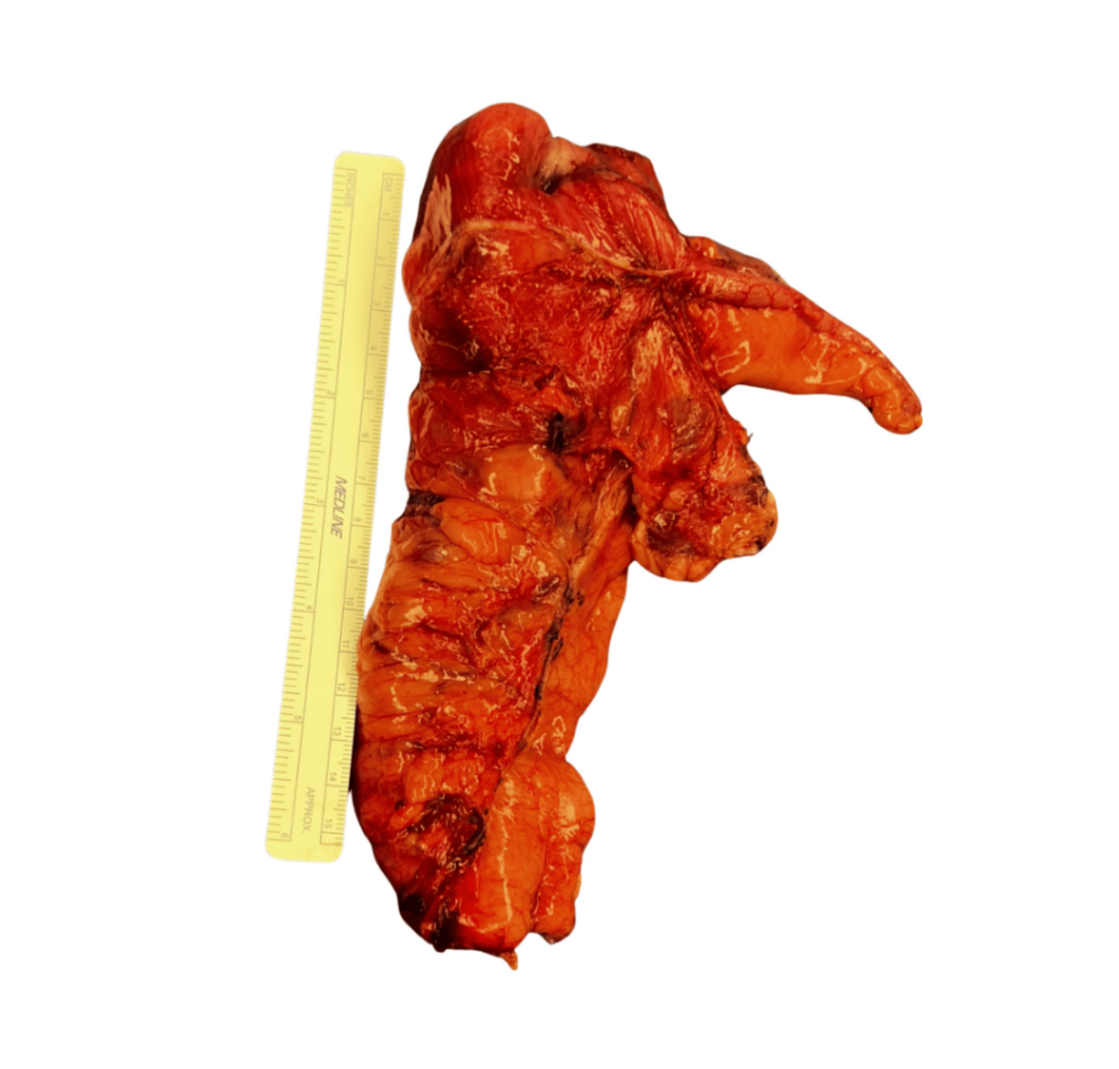
Gross intraoperative specimen of the right colon measuring approximately 4 cm.

A diagnostic colonoscopy performed to prepare the bowel for primary anastomosis and for endoscopical visualization for tissue biopsy, which was conducted on the second day of admission, revealed a large, friable, and exophytic cecal mass. Cold biopsies were obtained during the procedure. Immunophenotyping was performed to exclude other causes and demonstrated low MSI, intact DNA mismatch repair proteins MLH1, MSH2, MSH6, and PMS2, which signified the patient did not have microsatellite instability. Due to the high risk of malignancy-associated intussusception, surgical intervention was recommended. On the third day of admission, the patient underwent an exploratory laparotomy with oncologic right hemicolectomy with regional lymphadenectomy, where gross negative margins were confirmed with frozen sections intraoperatively (Figure 4). An open approach was favored due to a large palpable cecal mass with mesenteric lymphadenopathy and suspected hepatic involvement, allowing for optimal exposure to ensure thorough staging, safe oncologic resection, and adequate lymphadenectomy. Intraoperative frozen section confirmed that the tumor was confined to the cecum, and a more extensive resection was not pursued, as it was not expected to provide additional oncologic benefit.

**Figure 5:**
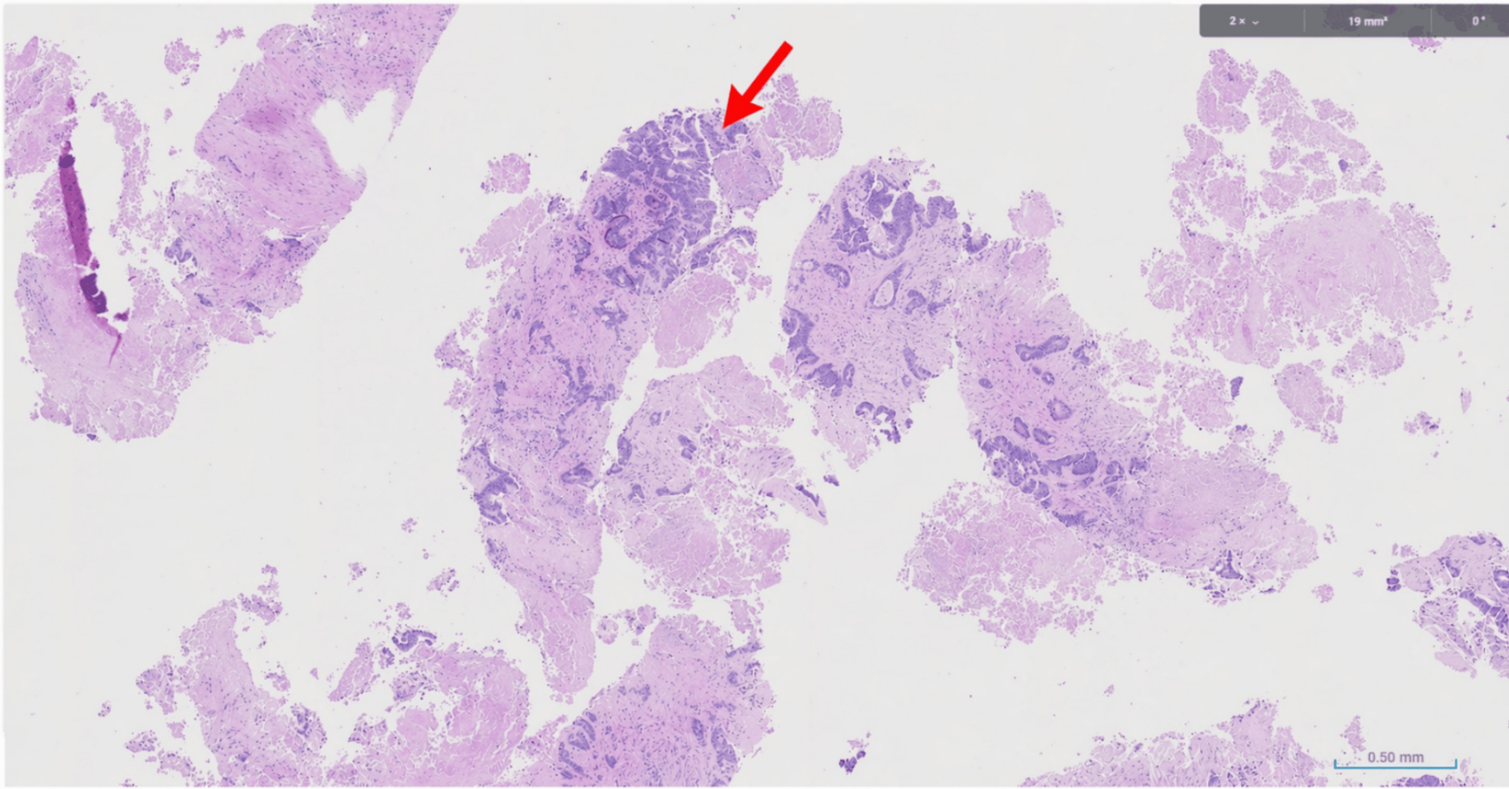
H&E-stained section of colonic mucosa showing moderately differentiated adenocarcinoma. Low-power hematoxylin and eosin (H&E)-stained section showing fragments of colonic mucosa with infiltrative, irregular gland-forming epithelium and surrounding desmoplastic stroma (red arrow), consistent with moderately differentiated adenocarcinoma.

**Figure 6:**
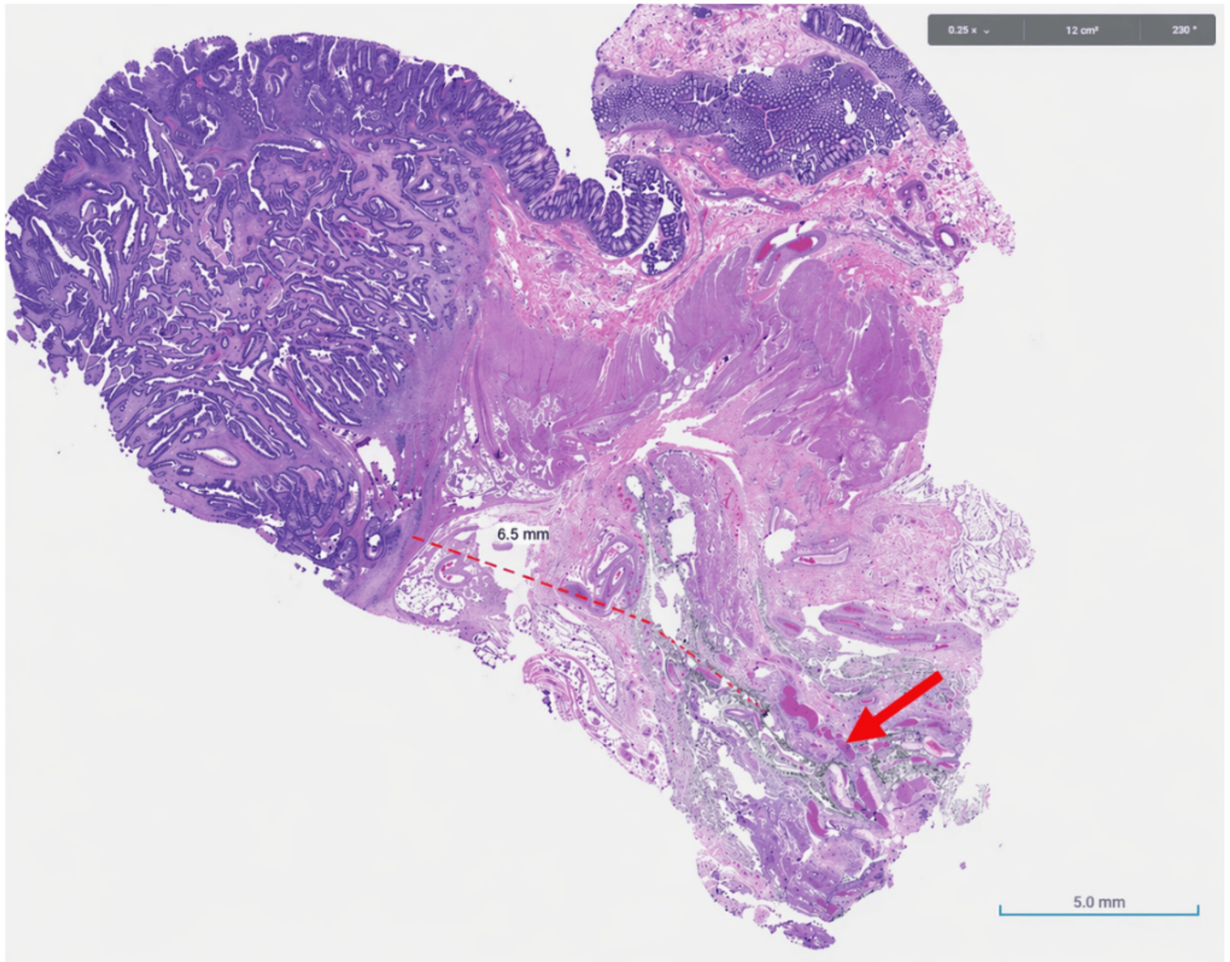
H&E-stained section of right hemicolectomy specimen showing invasive colonic adenocarcinoma. H&E section of right hemicolectomy specimen demonstrating adenocarcinoma arising in a tubulovillous adenoma with invasion through the muscularis propria into the pericolonic adipose tissue. The dashed line indicates approximately 6.5 mm depth of extramural invasion (pT3), and the red arrow marks infiltrative malignant glands within the desmoplastic stroma. H&E: hematoxylin and eosin

Postoperative recovery was uncomplicated, with a gradual return of bowel function and tolerance of oral intake. Final surgical pathology demonstrated a 4.0 cm moderately differentiated adenocarcinoma invading through the muscularis propria into the pericolonic tissue and appendix, with lymphovascular invasion (Figures 5, 6). Two of twenty regional lymph nodes were positive for metastatic disease, with negative surgical margins, corresponding to the pathologic stage pT3N1b.

Postoperative staging included a CT scan of the chest, which showed no pulmonary metastases. A magnetic resonance imaging (MRI) of the liver was inconclusive, prompting a CT-guided liver biopsy on the seventh day of admission, which confirmed metastatic adenocarcinoma consistent with colonic origin. A central venous port was placed for anticipated systemic chemotherapy, and the patient was referred for outpatient oncology follow-up, including positron emission tomography (PET)-CT and initiation of chemotherapy.

## Discussion

Adult intussusception is a rare clinical phenomenon, accounting for approximately 5% of all intussusception cases and 1-5% of adult bowel obstructions [8,9]. In contrast to pediatric cases, adult intussusception is usually secondary to an identifiable PLP, most commonly a neoplasm. In cases involving the colon, malignancy is the predominant etiology, with colorectal adenocarcinoma accounting for up to 60-65% of adult colonic intussusceptions. As a result, surgical exploration in adult patients is generally both diagnostic and therapeutic [9]. Mechanistically, in malignant cases, the tumor serves as a structural lead point within the bowel wall or lumen. This focal lesion alters normal peristaltic activity and becomes engaged by peristaltic waves, resulting in invagination of the proximal bowel into the adjacent distal segment. As the intussuscepted segment advances, compression of the mesentery may impair venous outflow, leading to bowel wall edema, progressive obstruction, ischemia, and, in severe cases, perforation. This contrasts with pediatric intussusception, which is typically idiopathic, whereas adult cases more often involve an identifiable lead point such as a benign or neoplastic lesion [8-10]. 

Cecal adenocarcinoma presenting as ileocolic intussusception is uncommon, particularly in young adults. While colorectal cancer incidence has historically increased with age, early-onset colorectal cancer, defined as diagnosis before the age of 45 years, is increasingly recognized. These patients often present with nonspecific symptoms, contributing to delayed diagnosis and more advanced disease at presentation, resulting in a worse prognosis [11-13].

This case is especially significant given the patient’s age of 31 years, well below standard screening thresholds, and the absence of a clear colorectal cancer family history. Such presentations highlight how early-onset colorectal cancer may occur outside traditional risk profiles, leading to lower clinical suspicion and diagnostic delay. The patient’s gastrointestinal symptoms, including intermittent abdominal pain, melena, and anemia, were nonspecific and lacked classic obstructive findings, further complicating timely recognition. This reflects a broader challenge, as the current United States Preventive Services Task Force guidelines recommend initiating screening at the age of 45 years for average-risk individuals, leaving younger patients more vulnerable to delayed diagnosis [14]. 

CT imaging of the abdomen and pelvis with intravenous contrast remains the gold standard diagnostic modality for adults with suspected small bowel obstruction, with reported sensitivities exceeding 80% [15,16]. Characteristic findings include the “target” sign or a “sausage-shaped” mass, and CT imaging may also identify a PLP or evidence of metastatic disease [17]. Surgical pathology following right hemicolectomy in our case confirmed moderately differentiated adenocarcinoma arising from a tubulovillous adenoma, with lymphovascular invasion and nodal metastases (pT3N1b). Immunohistochemical staining demonstrated intact expression of MLH1, MSH2, MSH6, and PMS2 proteins, consistent with microsatellite stability, not suggestive of Lynch syndrome, making the presentation even more rare. Surgical resection without attempted reduction is recommended for adult colonic intussusception due to the high likelihood of underlying malignancy and the risk of tumor dissemination or bowel perforation associated with reduction [18-19]. This patient appropriately underwent an open right hemicolectomy with resection of the terminal ileum, cecum, and appendix, including high ligation of the ileocolic pedicle and mesenteric lymphadenectomy, followed by a side-to-side ileocolic anastomosis. Intraoperatively, no peritoneal carcinomatosis was identified, and hepatic lesions were noted but deferred for percutaneous biopsy. The subsequent confirmation of hepatic metastases underscores the tendency of right-sided colorectal cancers to present at more advanced stages and to carry a poorer prognosis in comparison with left-sided tumors [20]. This case highlights the importance of maintaining a high index of suspicion for colorectal cancer in young adults presenting with bowel obstruction, anemia, or unexplained gastrointestinal symptoms. In that regard, early imaging, prompt surgical management, and coordinated multidisciplinary care remain essential for improving outcomes in these atypical and aggressive presentations.

## Conclusions

Clinical vigilance is warranted when evaluating younger patients with persistent or unexplained gastrointestinal symptoms, including abdominal pain, anemia, or gastrointestinal bleeding. Although colorectal cancer is less common in this population, atypical presentations, such as intussusception, may represent an underlying malignancy. 

Contrast-enhanced CT imaging plays a central role in diagnosis, while timely surgical intervention remains essential for both diagnosis and management. Although broader screening or hereditary implications cannot be established from a single case, this report underscores the need for heightened clinical suspicion for colorectal malignancy in younger patients with atypical presentations.
